# HIF-1α is a negative regulator of interferon regulatory factors: Implications for interferon production by hypoxic monocytes

**DOI:** 10.1073/pnas.2106017118

**Published:** 2021-06-09

**Authors:** Travis Peng, Shin-Yi Du, Myoungsun Son, Betty Diamond

**Affiliations:** ^a^Center for Autoimmune Musculoskeletal and Hematopoietic Diseases, Feinstein Institutes for Medical Research, Manhasset, NY 11030;; ^b^Department of Molecular Medicine, Donald and Barbara Zucker School of Medicine at Hofstra/Northwell, Hempstead, NY 11549

**Keywords:** HMGB1, hypoxia, HIF-1α, type I interferon, COVID-19

## Abstract

Patients with severe COVID-19 infection exhibit a low level of oxygen in affected tissue and blood. To understand the pathophysiology of COVID-19 infection, it is therefore necessary to understand cell function during hypoxia. We investigated aspects of human monocyte activation under hypoxic conditions. HMGB1 is an alarmin released by stressed cells. Under normoxic conditions, HMGB1 activates interferon regulatory factor (IRF)5 and nuclear factor-κB in monocytes, leading to expression of type I interferon (IFN) and inflammatory cytokines including tumor necrosis factor α, and interleukin 1β, respectively. When hypoxic monocytes are activated by HMGB1, they produce proinflammatory cytokines but fail to produce type I IFN. Hypoxia-inducible factor-1α, induced by hypoxia, functions as a direct transcriptional repressor of IRF5 and IRF3. As hypoxia is a stressor that induces secretion of HMGB1 by epithelial cells, hypoxia establishes a microenvironment that favors monocyte production of inflammatory cytokines but not IFN. These findings have implications for the pathogenesis of COVID-19.

## Significance

Clinical studies have shown that defects in type I interferon (IFN) production or antibodies to IFN appear to correlate with severe COVID-19 infection. Here, we demonstrate that proinflammatory cytokines but not type I IFN are produced by hypoxic human monocytes. We show that hypoxia suppresses production of type I IFN but not nuclear factor-κB–dependent proinflammatory cytokines. We demonstrate that hypoxia-inducible factor-1α is a direct transcriptional suppressor of interferon regulatory factors, the transcriptional activators of type I IFN. These findings may aid in understanding and control of impaired IFN production by severe acute respiratory syndrome coronavirus 2 infection.

COVID-19 most often presents as a respiratory infection with viral propagation in lung alveolar cells (1, 2). Severe infection is characterized by lung injury and extreme systemic hypoxia (3, 4). Monocytes are routinely recruited to sites of tissue injury. Increased accumulation of monocytes in injured tissue contributes to local hypoxia (5). Reciprocally, hypoxia in tissue leads to increased recruitment of monocytes and to their differentiation to monocyte-derived macrophages (6, 7). The hypoxic microenvironment forces cells to shift their metabolism from oxidative phosphorylation to anaerobic glycolysis and leads to chromatin remodeling and a new transcriptional program (8, 9). Strikingly, hypoxia and inflammatory triggers induce some shared transcriptional programs in monocytes, including the activation of members of both the hypoxia-inducible factor (HIF) and nuclear factor-κB (NF-κB) families (10, 11). Although inflammatory monocytes in normoxic environments have been studied extensively, there is little information on how hypoxia alters monocytes recruited to tissue in the course of an infection or inflammatory process.

HIF family members function as transcriptional regulators of hypoxia (12–14). Under hypoxic conditions, HIF-1α subunits are stabilized and accumulate in the nucleus where they dimerize with HIF-1β, allowing them to bind to DNA and stimulate the transcription of their target genes (15). HIFs bind the core consensus sequence 5′-(A/G)CGTG-3′ within the hypoxia-response element (HRE) present in many genes (16–18). It is well-appreciated that HIF-1α induces various genes involved in metabolism such as the glucose transporter GLUT1, lactate dehydrogenase (LDH), which catalyzes lactate production from pyruvate, and pyruvate dehydrogenase kinase (PDK1), which inhibits pyruvate dehydrogenase, thereby maintaining high levels of pyruvate (14, 19). This altered metabolic program helps increase HIF-1α protein levels, as accumulation of lactate and pyruvate have been found to result in HIF-1α stabilization (20–22). Succinate, induced in the same metabolic pathway, also stabilizes HIF-1α and drives inflammation through enhanced production of interleukin (IL)-1β (23).

High mobility group box 1 (HMGB1) acts as an alarmin in damaged tissue as it can be secreted by activated immune cells recruited to the site of injury or passively released by stressed tissue-resident cells (24, 25). HMGB1 has been shown to be present in a hypoxic environment (24). Because HMGB1 binds both DNA and RNA and transports these to intracellular toll-like receptors (TLRs) 7 and 9 in a RAGE-dependent fashion, it triggers an inflammatory response (26). HMGB1-stimulated monocytes secrete proinflammatory cytokines and type I interferon (IFN) through an MyD88-IRF5–dependent pathway (27–29). As NF-κB activation is enhanced by interferon regulatory factor (IRF)5, IRF5 has been considered to be a master regulator of inflammatory macrophages. Consistent with this paradigm, we have previously demonstrated suppression of IRF5 under normoxic conditions decreases production of both type I IFN and proinflammatory cytokines (30, 31). While several studies have shown that type I IFN is reduced in hypoxic conditions, the detailed mechanism for this is not known and upstream factors that may modulate the expression or activity of IRF5 have not been well-studied.

Here, we asked how hypoxia alters the response to the alarmin HMGB1. We demonstrate that monocytes exposed to HMGB1 under hypoxic conditions or after exposure to dimethyloxaloylglycine (DMOG), which increases levels of HIF-1α, exhibit decreased IRF5 expression and decreased production of type 1 IFN while still exhibiting activation of NF-κB and downstream cytokines. The suppression of IRF5 under hypoxic conditions is HIF-1α–dependent, as HIF-1α is a direct transcriptional suppressor of IRF5. We further show release of HMGB1 by epithelial cells stressed by hypoxia. Hypoxia-induced HIF-1α alters monocyte cellular metabolism, suppresses type I IFN, and releases an inflammatory program that is independent of IRF5.

## Results

### Hypoxia Alters the Monocyte Response to HMGB1.

**Fig. 1.**
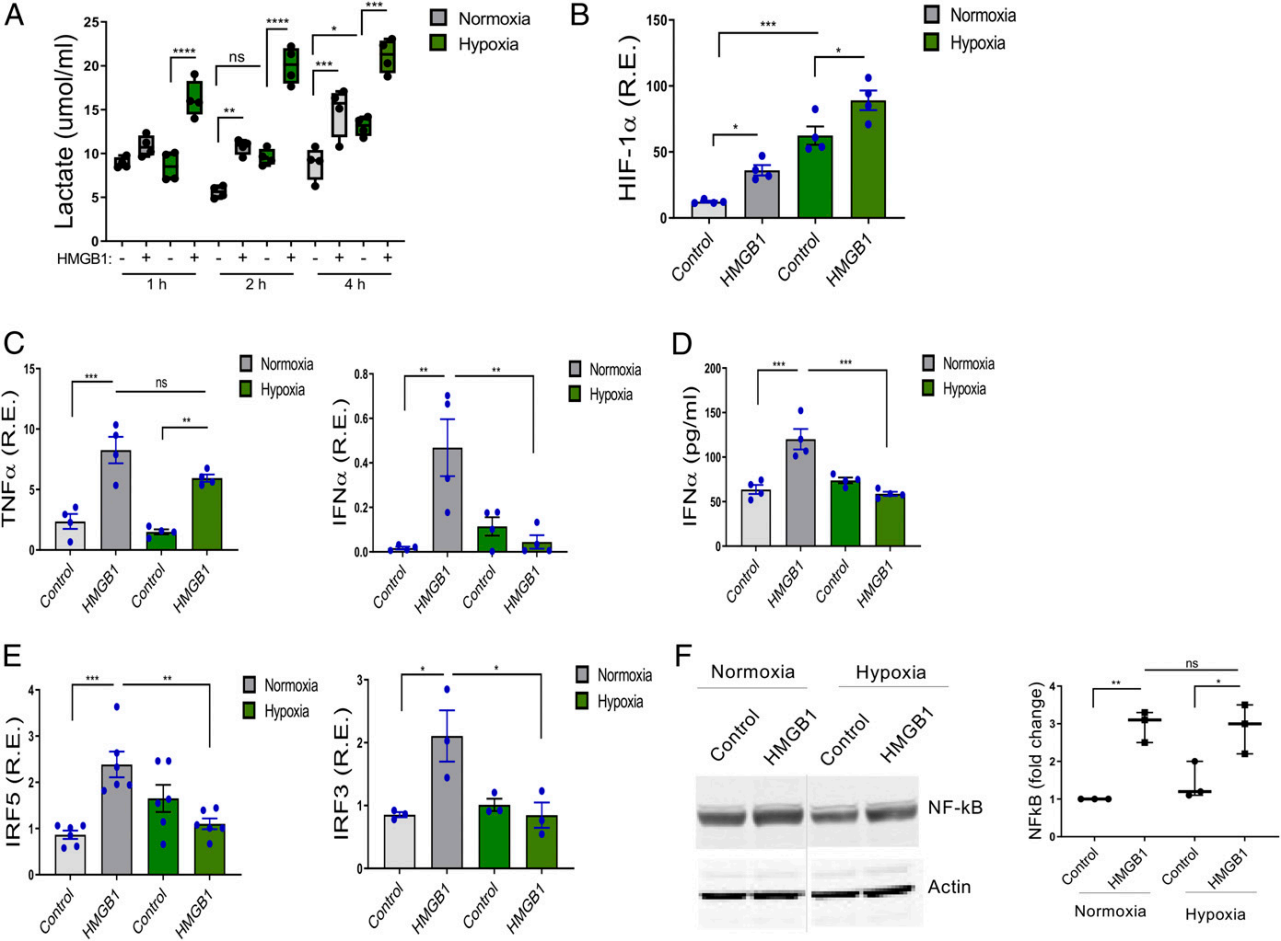
Hypoxia reduces HMGB1-induced IRF5, IRF3, and IFNα but not TNFα. (*A*) Human monocytes were exposed to 21% (normoxia) and 2% (hypoxia) oxygen with or without HMGB1 (1 μg/mL). Lactate measured from cell supernatants at indicated time points demonstrated significantly increased lactate in cells exposed to hypoxia and HMGB1. Floating bars (minimum to maximum), the line at median, each symbol represents an individual experiment in triplicate, *n* = 4. (*B*–*D*) The expression of the HIF-1α, TNFα, IFNα, IRF5, and IRF3 were analyzed by qRT-PCR (4 h). Data represent mean ± SEM of four independent experiments in which each condition was tested in triplicate. (*E*) The level of IFNα secretion was determined by ELISA from cell supernatant at 24 h. *n* = 4. (*F*) NF-κB was induced by HMGB1 in both normoxic and hypoxic cells. NF-κB was analyzed by Western blots in cells under resting and HMGB1-stimulated, normoxic and hypoxic conditions. One representative of three independent experiments (*Left*). Fold changes compared to normoxic control were calculated by band intensity (*Right*). The lanes were run on the same gel but were noncontiguous (dotted line). *n* = 3. Each symbol represents an individual experiment. One-way ANOVA *P ≤ 0.05; **P ≤ 0.01; ***P ≤ 0.001; ns, *P* > 0.05.

To confirm a hypoxic response in human monocytes exposed to 2% O_2_, we demonstrated increased lactate compared to monocytes maintained in normoxic conditions (Fig. 1*A*). Because HMGB1 can be present in a hypoxic environment, we assessed the impact of HMGB1 stimulation on lactate production by normoxic and hypoxic monocytes after 4 h of incubation. HMGB1 induced lactate production in both normoxic and hypoxic monocytes. There was, however, greater lactate production in hypoxic cells (Fig. 1*A*), demonstrating that both hypoxia and HMGB1 stimulation alter monocyte metabolism. This was evident at 1 h, before transcriptional changes are detectable, and sustained for at least 4 h. We also observed an increase in succinate after 4 h of hypoxia, as well as an HMGB1-driven increase in succinate at 2 and 4 h. Pyruvate was induced by 2 and 4 h of hypoxia, but not by HMGB1 (*SI Appendix*, Fig. S1).

To further confirm that 2% O_2_ for 4 h induced a hypoxic program, we measured HIF-1α expression in monocytes exposed to 2% O_2_ for 4 h and in normoxic monocytes, with or without exposure to HMGB1. We observed an increase in HIF-1α expression in monocytes grown at low O_2_ levels. HMGB1 stimulation also induced HIF-1α expression, such that HIF-1α expression was highest in HMGB1-stimulated cells under hypoxic conditions (Fig. 1*B*). To further explore the effects of HMGB1 on hypoxic monocytes, we measured induction tumor necrosis factor α (TNFα) and IFNα, both of which are induced by HMGB1 in normoxic monocytes. TNFα was induced by HMGB1 in hypoxic conditions but IFNα was not (Fig. 1 *C* and *D*). HMGB1 induced IRF5 or IRF3 under normoxic conditions (Fig. 1*E*). Consistent with the absence of IFNα induction, HMGB1 did not induce IRF5 and IRF3 in hypoxic monocytes (Fig. 1*E*); in contrast, NF-κB was induced by HMGB1 in both normoxic and hypoxic cells (Fig. 1*F*), and there was no difference in NF-κB induction by HMGB1 between normoxic and hypoxic monocytes. Thus, HMGB1 stimulation under hypoxic conditions suppressed IRF5 and IRF3, but not NF-κB, leading to a cytokine response lacking IFNα. Besides inflammatory monocytes, plasmacytoid dendritic cells (pDCs) and macrophages are producers of type I IFN (32). Monocyte-derived macrophages increased expression of TNFα and IRF5 and increased secretion of IFNα after exposure to HMGB1 under normoxic conditions but exhibited increased expression of TNFα, decreased expression of IRF5, and decreased secretion of IFNα after exposure to HMGB1 under hypoxic conditions (*SI Appendix*, Fig. S2 *A* and *C*). Because pDCs are the cells that produce the greatest amount of IFNα on a per-cell basis, we also analyzed pDCs with or without exposure to HMGB1 under normoxic or hypoxic conditions. PDCs also showed decreased expression of IRF5 and decreased IFNα secretion after exposure to HMGB1 under hypoxic conditions (*SI Appendix*, Fig. S2 *B* and *C*).

### DMOG Promotes HMGB1-Induced NF-κB but Inhibits IRF5.

**Fig. 2.**
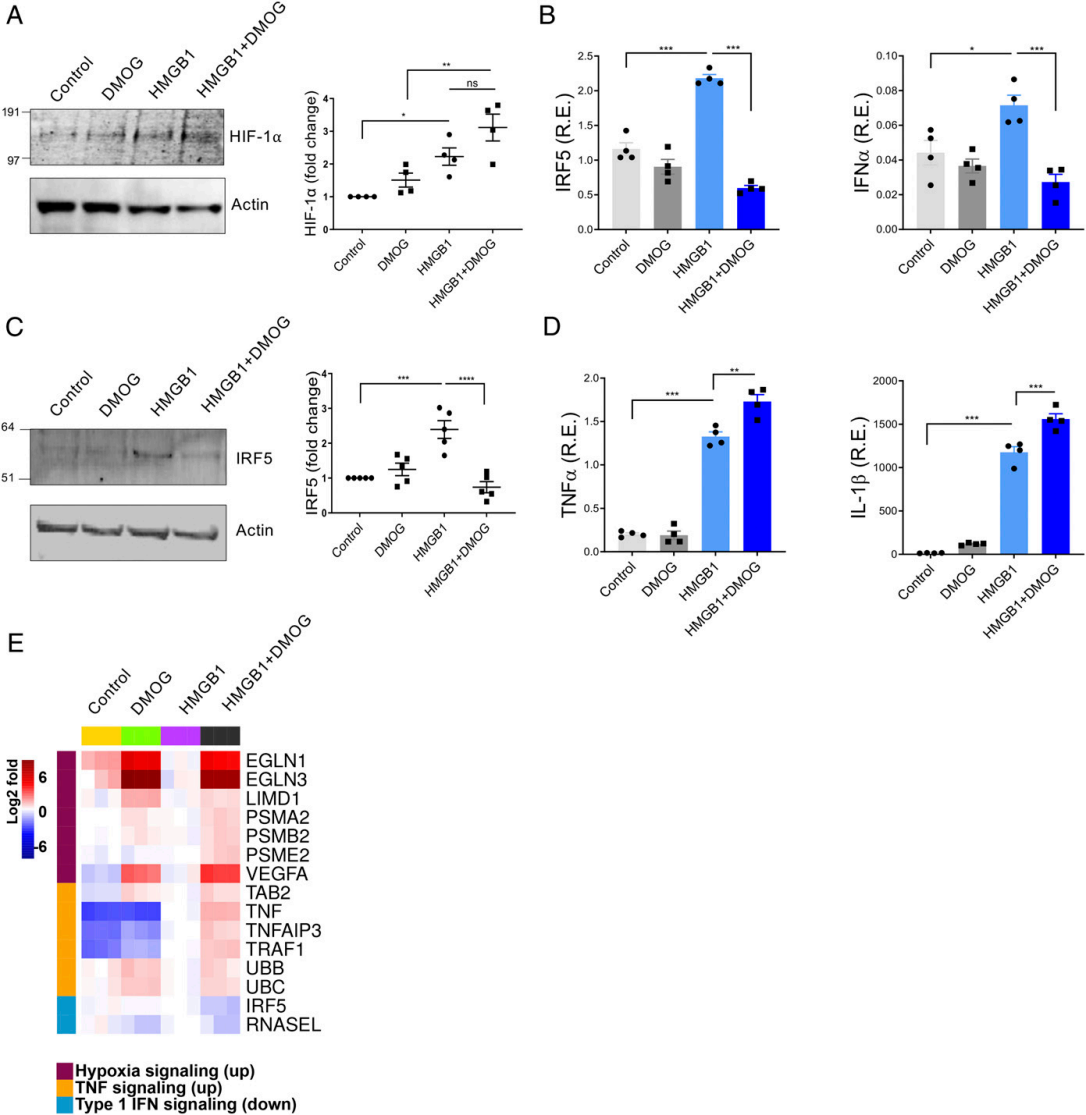
DMOG and HMGB1 down-regulate IRF5 and IFN signaling and mimic hypoxia conditions. Human monocytes were preincubated with DMOG (25 μM) for 1 h and stimulated with HMGB1 (1 μg/mL) for 4 h. Each condition was tested in triplicate. (*A*) Western blots analyzed the level of HIF-1α. One representative of four independent experiments (*Left*). Fold changes compared to control were calculated by band intensity (*Right*). (*B*) IRF5 or IFNα mRNA. Mean ± SEM, *n* = 4 (*C*) IRF5 protein. Fold changes compared to untreated control were calculated by band intensity. Mean ± SEM, *n* = 5. (*D*) TNFα or IL-1β mRNA. Mean ± SEM, *n* = 4. One-way ANOVA. *P ≤ 0.05; **P ≤ 0.01; ***P ≤ 0.001; ns, *P* > 0.05. (*E*) A heat map generated from RNA-seq of control, DMOG-, HMGB1-, and HMGB1 plus DMOG–treated monocytes (4 h). Two donors were analyzed in triplicate.

Prolyl-4-hydroxylases (PHDs) hydroxylate HIFs for rapid destruction by proteasomal degradation following ubiquitinylation (33). DMOG is a cell-permeable prolyl-4-hydroxylase inhibitor which up-regulates HIF-1α (34–36). DMOG can decrease cellular respiration (35), and as such it has been used as a surrogate for hypoxia. We, therefore, asked whether exposure to DMOG would induce the same transcriptional program as hypoxia. While exposure to DMOG did not increase HIF-1α expression, exposure to DMOG and HMGB1 led to increased HIF-1α compared to exposure to HMGB1 in the absence of DMOG (Fig. 2*A*). We next confirmed that DMOG exposure led to increased lactate, which was further increased by exposure to DMOG and HMGB1 (*SI Appendix*, Fig. S3*A*). We then examined IRF5 and IFNα transcript levels. In the presence of HMGB1, IRF5 and IFNα messenger RNA (mRNA) expression was increased as expected; when DMOG was present, IRF5 and IFNα levels did not increase with HMGB1 stimulation (Fig. 2*B*). HMGB1-induced IRF5 protein levels were also decreased by DMOG (Fig. 2*C*). In contrast, expression of the proinflammatory cytokines, TNFα and IL-1β, was increased in HMGB1-treated cells and further increased when DMOG was present (Fig. 2*D*). Unlike IRF5, NF-κB expression was increased by either HMGB1 or DMOG exposure (*SI Appendix*, Fig. S3*B*). We also observed that DMOG reduced IRF3 transcription induced by HMGB1, while other IRFs, IRF4 and IRF7, did not change in response to HMGB1 or DMOG in human monocytes (*SI Appendix*, Fig. S3*C*). RNA sequencing (RNA-seq) of human monocytes stimulated by HMGB1 in the absence or presence of DMOG confirmed these results, showing an up-regulated hypoxia signal when DMOG was present and increased TNF signaling in the presence of HMGB1 that was further enhanced when HMGB1 and DMOG were both present. Down-regulated IRF5 and type I IFN signaling were observed in the presence of HMGB1 and DMOG (Fig. 2*F*). These results demonstrate that HMGB1 does not activate IRF5 or IFNα when HIF-1α levels are increased, but the HMGB1-induced activation of NF-κB and proinflammatory cytokine in RNA is not diminished by HIF-1α.

### HIF-1α Suppresses IRF5 and IRF3 Transcription.

**Fig. 3.**
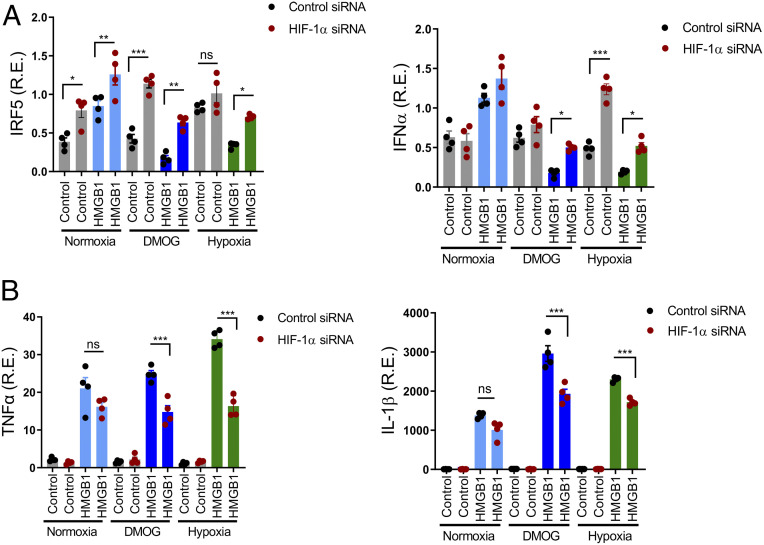
IRF5 and IFNα repression in response to HMGB1 and hypoxia are HIF-1α-dependent. (*A* and *B*) Human monocytes were transfected with control or HIF-1α siRNA. Twenty-four hours after transfection, cells were stimulated with DMOG or HMGB1 plus DMOG for 4 h. For hypoxic conditions, cells were cultured in 2% O_2_ for 4 h. IRF5, IFNα, TNFα, and IL-1β transcripts were determined by qRT-PCR, mean ± SEM, *n* = 4. One-way ANOVA. *P ≤ 0.05; **P ≤ 0.01; ***P ≤ 0.001; ns, *P* > 0.05.

While an association of hypoxia and low type I IFN has been shown in cancer (37) the mechanism has not been determined. To ask if the low IRF5 expression in hypoxic or DMOG-treated cells was caused by increased HIF-1α, we transfected primary monocytes with control or HIF-1α small interfering RNA (siRNA) and exposed them to normoxic or hypoxic conditions or to DMOG. HIF-1α siRNA-transfected cells showed higher IRF5 in all culture conditions than control siRNA-transfected cells (Fig. 3 *A*, *Left*). Moreover, the reduction of IRF5 by HMGB1 and hypoxia was reversed in HIF-1α siRNA-transfected cells. IFNα mRNA levels also correlated with IRF5 levels (Fig. 3 *A*, *Right*). Interestingly, HIF-1α siRNA-transfected cells showed lower TNF and IL-1β mRNA expression after HMGB1 stimulation in hypoxic conditions, confirming a role for HIF-1α in NF-κB activation (Fig. 3*B*). Thus, HIF-1α is a positive regulator of proinflammatory cytokines and a negative regulator of IRF5 and may be a direct transcriptional repressor of IRF5.

**Fig. 4.**
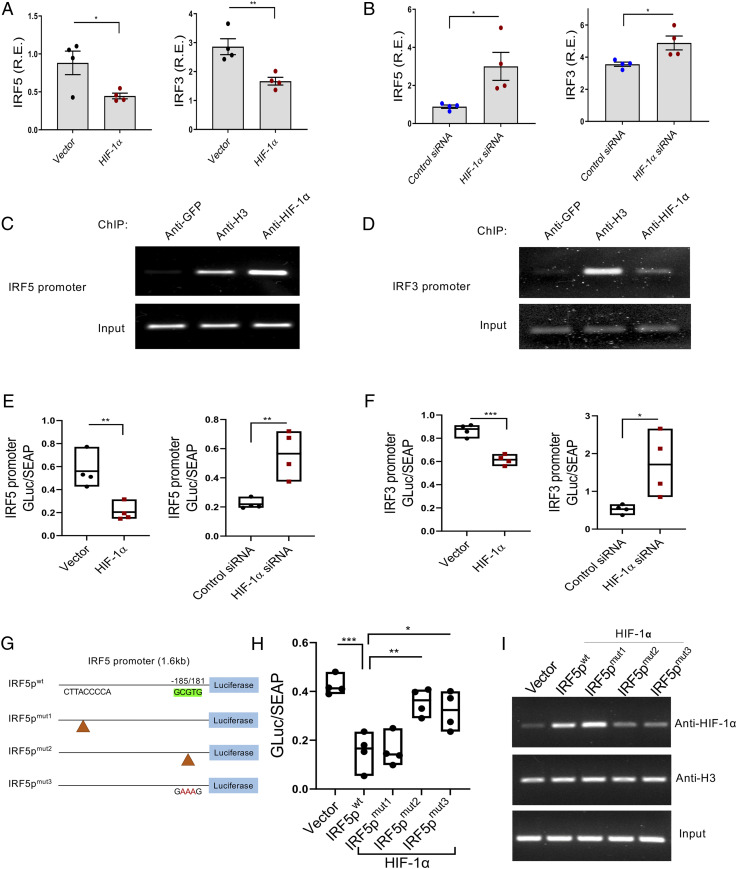
HIF-1α is a transcriptional repressor of IRF5 and IRF3. (*A* and *B*) HIF-1α down-regulated IRF5 and IRF3 transcripts in human monocytes. HIF-1α encoding plasmid or HIF-1α siRNA was transfected into primary human monocytes and IRF5 or IRF3 mRNA was measured 24 h after transfection, mean ± SEM, *n* = 4. Unpaired *t* test. *P ≤ 0.05; **P ≤ 0.001. (*C* and *D*) HIF-1α binds the IRF5 promoter and IRF3 promoter. ChIP assay coupled to PCR was performed in HIF-1α–overexpressing monocytes. Anti-GFP, histone H3, and HIF-1α antibodies and primers covering the putative HIF-1α binding site on the IRF5 or IRF3 promoter regions were used. Data represent one of the three independent experiments. (*E* and *F*) IRF5 or IRF3-promoter constructs and HIF-1α–encoding plasmid or HIF1α siRNA were transfected into human monocytes. Gaussia luciferase (Gluc) and SEAP activities were measured from the cell supernatant (24 h). The ratio of luciferase to SEAP was calculated. Floating bars (minimum to maximum), each symbol represents an individual experiment. Unpaired *t* test. *P ≤ 0.05; **P ≤ 0.01; ***P ≤ 0.001. *n* = 4. (*G*) Schematic of HIF1α binding site on IRF5 promoter and the constructed mutants. (*H*) The luciferase assay determined that HIF1α suppressed the IRF5 promoter through the HRE, GCGTG. The ratio of luciferase to SEAP was measured at 24 h. *P ≤ 0.05; **P ≤ 0.01; ***P ≤ 0.001. *n* = 4. (*I*) HIF-1α binds wild-type IRF5 promoter (IRF5p^wt^) and IRF5 promoter mutant 1 (IRF5p^mut1^) but not HRE mutants of the IRF5 promoter (IRF5p^mut2^ and IRF5p^mut3^). The transient ChIP assay from HEK293T cells was performed with the mutant constructs. Data represent one of the three independent experiments.

HIFs activate hypoxia-responsive genes through binding to the HRE consensus sequence 5′-(A/G)CGTG-3′ in the promoter region (38). We investigated the promoter regions of IRF5 and IRF3 and found potential HIF-1α binding sites in the 5′-end of both (*SI Appendix*, Fig. S4 *A* and *B*). To see if HIF-1α affected transcription of IRF5 and IRF3, human monocytes were transfected with an HIF-1α–expressing plasmid or HIF-1α siRNA. IRF5 and IRF3 transcripts were lower in HIF-1α–overexpressing cells and higher in HIF-1α siRNA-transfected cells than in control cells (Fig. 4 *A* and *B*). To further assess whether HIF-1α might repress IRF5 or IRF3 by direct binding to the promoter region, we performed a chromatin immunoprecipitation (ChIP) assay. After 24 h of transfection with HIF-1α or control vector, nuclear lysates from monocytes were cross-linked, sonicated, and immunoprecipitated with antibodies specific to HIF-1α, green fluorescent protein (GFP) (as a negative control), or histone H3 (as a positive control). As expected, PCR products were observed in anti-H3 antibody but not in anti-GFP antibody immunoprecipitated conditions. Anti–HIF-1α antibody immunoprecipitated both IRF5 and IRF3 (Fig. 4 *C* and *D*). Using Gaussia luciferase reporter constructs with either an IRF5 or an IRF3 promoter, we measured luciferase activity in the supernatant after 24 h of transfection with a control vector or HIF-1α. Secreted alkaline phosphatase (SEAP) activity was used for normalization. Both IRF5 and IRF3 luciferase activity was significantly decreased in HIF-1α–overexpressing cells; in contrast, luciferase activity was increased in HIF-1α siRNA-transfected cells (Fig. 4 *E* and *F*). The same results were obtained in an HIF-1α–overexpressing HEK293T cell line (*SI Appendix*, Fig. S4*C*). To confirm that HRE regions are critical for HIF-1α binding and function, three sets of mutants were made in the promoter constructs by site-directed mutagenesis; CTTACCCCA was deleted (IRF5p^mut1^) and GCGTG was deleted (IRF5p^mut2^) or mutated to GAAAG (IRF5p^mut3^) (Fig. 4*G*). While HIF-1α suppressed the luciferase activity of IRF5p^wt^ and IRF5p^mut1^, HIF-1α did not suppress the IRF5p^mut2^ and IRF5p^mut3^ in either in monocytes (Fig. 4*H*) or HEK293T cells (*SI Appendix*, Fig. S4*D*). We confirmed this result with a ChIP assay (39). Using HEK293T cells transfected with each construct, IRF5p^mut2^ and IRF5p^mut3^ showed less HIF-1α binding than IRF5p^wt^ or IRF5p^mut1^ (Fig. 4*I*). These results provided direct evidence that HIF-1α binds to HRE in the IRF5 promoter region.

### A Hypoxic Microenvironment Induces HMGB1 Secretion.

**Fig. 5.**
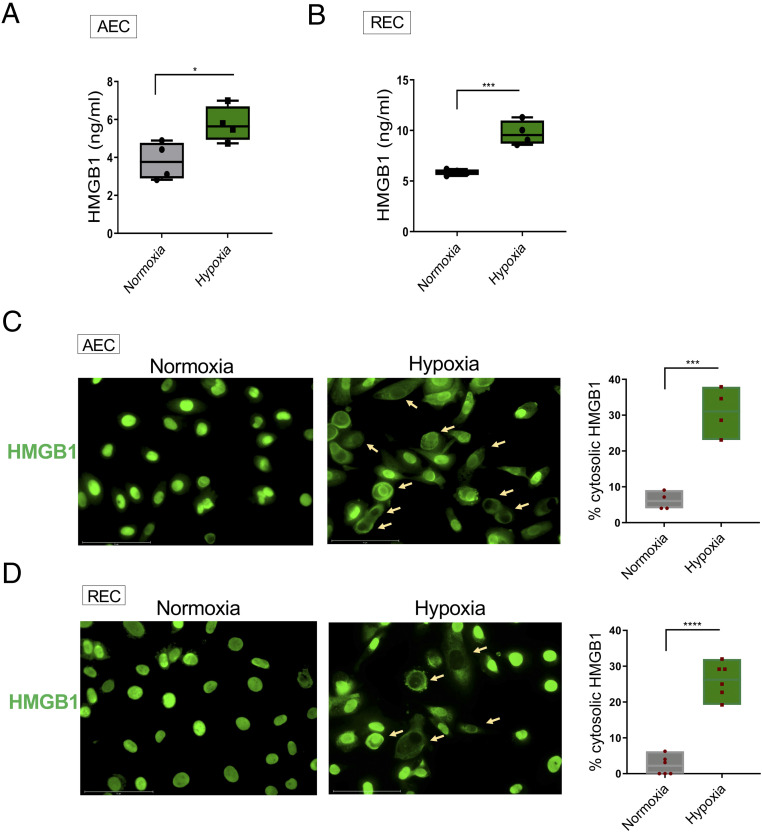
Hypoxia-induced HMGB1 release. (*A*) Lung alveolar epithelial A549 cells (AEC) were exposed to 21% (normoxia) or 2% (hypoxia) oxygen for 24 h and HMGB1 levels in supernatant were measured by ELISA. Floating bars (minimum to maximum), line at median, each symbol represents an individual experiment in triplicate, *n* = 4. (*B*) Primary renal tubular epithelial cells (REC) were exposed to normoxia or hypoxia for 48 h and HMGB1 levels in the supernatant were measured by ELISA, *n* = 4. (*C* and *D*) Hypoxia induces translocation of HMGB1 in AEC (24 h) and REC (48 h). Cells were stained with anti-HMGB1 antibody followed by Alexa Fluor 488–conjugated secondary antibody. Representative fluorescence microscopic images from three independent experiments are shown (original magnification, ×40). The percentage of cells with cytosolic HMGB1 (arrows) were counted. Unpaired *t* test. *P ≤ 0.05; ***P ≤ 0.001; ****P ≤ 0.0001. (Scale bars, 75 μm.)

We next asked whether hypoxia in tissue might expose infiltrating monocytes to HMGB1. We incubated the A549 human lung epithelial cell line (AEC) and primary renal epithelial cells (REC) in normoxic or hypoxic conditions in serum-free medium. HMGB1 levels in culture supernatants were increased in hypoxic compared to normoxic cultures (Fig. 5 *A* and *B*). Consistent with this observation, higher levels of cytoplasmic HMGB1 were detected in hypoxia-exposed cells than normoxia-exposed cells (Fig. 5 *C* and *D*). Since HMGB1 will be present in the supernatant of dying cells, apoptosis was assessed by immunostaining for activated caspase-3. No difference in apoptosis was observed (*SI Appendix*, Fig. S5). Furthermore, there was no difference in cell viability, assessed by trypan blue exclusion (98 vs. 97% viable in normoxic and hypoxic AEC, 97 vs. 95% viable in normoxic and hypoxic conditions). These results demonstrate that hypoxia causes HMGB1 to be released from epithelial cells and suggest that HMGB1 will be present in hypoxic tissue to modulate monocyte metabolism, cytokine production, and activation state.

## Discussion

**Fig. 6.**
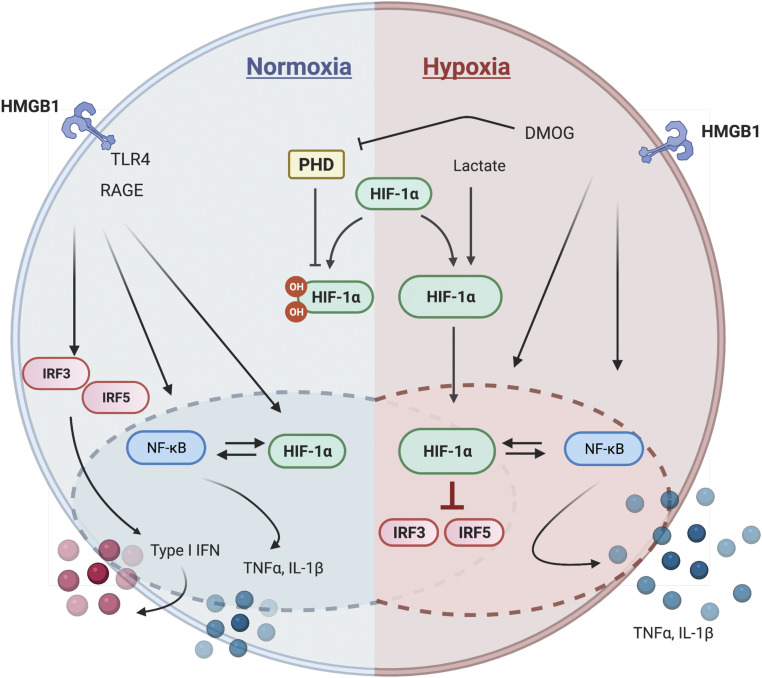
Schematic representation of HIF-1α’s role in hypoxic inflammation. HMGB1-exposed primary monocytes release proinflammatory cytokines and type I IFN through activating NF-κB and IRF3 and 5 (*Left*). In hypoxic conditions or following DMOG treatment only NF-κB is activated as HIF-1α represses IRF3 and IRF5 (*Right*). Thus, increased HIF-1α leads to production of proinflammatory cytokines but not IFN.

Clinical studies have shown that poor production of IFN can lead to severe disease in patients with COVID-19 and that individuals with antibodies to type I IFN also develop increased disease severity (40, 41). Respiratory viruses including severe acute respiratory syndrome coronavirus 2 (SARS-CoV-2) and influenza A virus encode antagonists to the IFN response (42–45). In this study, we identify another potential mechanism for low IFN levels in COVID-19 patients. Elevated levels of HIF-1α in primary human monocytes, induced by either hypoxia or DMOG exposure, lead to an altered response to HMGB1 activation characterized by activation of NF-κB and downstream proinflammatory cytokines but low levels of IRF5 secondary to direct transcriptional repression by HIF-1α, leading therefore to low levels to type I IFN (Fig. 6). The metabolic changes induced by hypoxia are likely responsible, in part, for the elevation of HIF-1α as it is established that metabolites such as lactate, succinate, and pyruvate lead to increased HIF-1α stabilization (46).

In normoxic conditions, HMGB1 is internalized by monocytes with its nucleic acid cargo and activates intracellular TLRs, leading to proinflammatory cytokine and type I IFN production (26, 35). IRF5 enhances NF-κB activity in normoxic conditions (47). In sharp contrast, under hypoxic conditions exposure to HMGB1 leads to an increased NF-κB activation pathway but reduced IRF5 and its downstream pathway. The exact mechanism for the enhanced NF-κB activation remains unclear, but activation of IκB kinase by inhibition of PHDs has been reported, as has activation of NF-κB by factor-inhibiting HIF (48). In HMGB1-stimulated monocytes, secretion of TNF may also induce an autocrine pathway to NF-κB1 activation. Moreover, hypoxia leads to succinate production, which in turn leads to IL1β release; IL1β, like TNF, activates NF-κB (23). Thus, in hypoxic HMGB1-stimulated monocytes, the increased NF-κB we observe may reflect a convergence of multiple pathways. As NF-κB also enhances transcription of HIF-1α, the transcriptional program in hypoxic monocytes is self-sustaining, with NF-κB enhancing HIF-1α and HIF-1α enhancing NF-κB.

We have shown that HIF-1α acts as a transcriptional repressor of IRF5 and IRF3. Of note, it has previously been shown that HIF-1α can act as a transcriptional repressor (18, 49, 50). The mechanisms for this are incompletely understood but include preventing the binding of transcriptional activators. This may not, however, be the only mechanism for low IRF5. Monocytes have developed specific regulatory programs that control TLR access to different endosomal compartments (51, 52). It is tempting to speculate, therefore, that the trafficking of TLRs to endosomes vs. lysosomes may also be altered by hypoxia and be another possible driver of the IRF5 suppression that occurs in the hypoxic inflammatory microenvironment.

It has been reported that IRF5 is a signature transcription factor that promotes M1 macrophage polarization and regulates glycolysis in macrophages (53, 54). HMGB1 is known to polarize monocytes into an M1-like macrophage phenotype with both IRF5 and NF-κB activation dependent on IRF5 (55, 56). Our results complicate the paradigm as we show that HMGB1 in hypoxia activates glycolysis and NF-κB pathways in a manner independent of IRF5. Why NF-κB depends on IRF5 in normoxia, but not hypoxia, requires exploration.

It has previously been reported that hypoxia diminishes the antitumor response by decreasing monocyte production of IRF3, resulting in type I IFN production (37). This diminishes activation of CD8^+^ cytolytic T cells, a process that is likely also operative in viral infections (57–59). We also found hypoxia decreased IRF3 and demonstrated that transcription of IRF3, like IRF5, is directly repressed by HIF-1α.

Strikingly, current studies demonstrate an impaired type I IFN response associated with severe and critical COVID-19 patients, which inversely correlates with an excessive NF-κB–driven inflammatory response associated with increased TNFα and other inflammatory cytokines. This has been attributed to genetic causes or to anti-IFN antibodies (40, 41). Our data suggest another mechanism for low IFN that is not genetically determined and not the result of antibodies but is a consequence of monocyte exposure to hypoxia and HMGB1. Indeed, a transcriptomic analysis of lung tissue from COVID-19 patients found up-regulation of HIF-1α and inflammatory cytokines but not type I IFN (60). We found that a lung epithelial cell line and primary renal epithelial cells release HMGB1 in hypoxic conditions. This requires confirmation in biopsy or autopsy tissue, but it strongly suggests that elevated levels of HMGB1 are likely to be present in hypoxic tissue and will activate the infiltrating monocytes. A study of respiratory syncytial virus provides evidence of HMGB1 involvement in lung inflammation (61), buttressing this model. As HIF-1α is induced in monocytes by hypoxia, and HMGB1 is released by hypoxic epithelial cells, these findings demonstrate that monocytes adapt to a local hypoxic environment with the remodeling of effector as well as metabolic pathways and that in COVID-19 patients the altered effector pathway is characterized by diminished production of IFNα, which contributes to disease pathogenesis. PDCs and macrophages secrete type I IFN and TNFα in inflammation under normoxia. We found that both pDCs and monocyte-derived macrophages, like monocytes, showed less induction of IRF5 and IFNα by HMGB1 in hypoxic conditions.

Our results, therefore, support a framework whereby an inflammatory cascade may be initiated by SARS-CoV-2 leading to elevated levels of HMGB1 that under hypoxic conditions trigger monocytes to make inflammatory cytokines but suppress type I IFN. Interestingly, HMGB1 has also been shown to function in a cell-intrinsic fashion to enable transcription of ACE-2, the cellular receptor for SARS-CoV-2 spike protein (62). Thus, HMGB1 may occupy a central place in COVID-19 pathology, both as an extracellular alarmin and as an intracellular regulator of transcription.

Overall, this study demonstrates that hypoxia leads to metabolic changes and alterations in the transcriptional programs and effector status of monocytes (Fig. 6). Hypoxia leads to an increased dependence on NF-κB and decreased dependence on IRF5, which is what is seen in COVID-19 patients. Thus, suppressed type I IFN is a consequence of the disease pathology and may be present in patients with tissue injury and hypoxia, not just in those with a genetic predisposition to low IFN production.

## Materials and Methods

### Human Monocyte Isolation and Cell Culture.

The protocol for studying of human samples was approved by the Institutional Review Board of the Feinstein Institutes for Medical Research. Peripheral blood mononuclear cells were isolated from the blood of deidentified healthy donors (New York Blood Center). CD14^+^CD16^−^ monocytes were further isolated using a human monocyte enrichment kit (Stem Cell Technology, 19059); purity of >95% was determined by flow cytometry (LSRII, BD Bioscience) using anti-human CD14 antibody (Thermo Fisher Scientific, 50-112-4936) (56). Purified monocytes (1 × 10^6^ cells/mL) were incubated in X-Vivo 15 serum free medium (Lonza, 04-418Q). Lung Epithelial A549 cells (ATCC, CCL-185) were maintained in F-12K Medium (ATCC, 30-2004) with 10% fetal bovine serum (FBS) and 1% PenStrep. Human primary renal tubular epithelial cells (ATCC, PCS400010) were grown in Renal Epithelial Cell Basal Medium (ATCC, PCS400030) supplemented with Renal Epithelial Cell Growth kit (ATCC, PCS400040). Renal Epithelial Cell Growth kit contains 0.5% FBS, 10 nM triiodothyronine, 10 ng/mL rh EGF, 100 ng/mL hydrocortisone hemisuccinate, 5 μg/mL rh insulin, 1 μM epinephrine, 5 μg/mL transferrin, and 2.4 mM lalanyl-l-glutamine. For subculture, cells were washed with DPBS (Life Technologies, 14190) and treated trypsin EDTA (ethylenediaminetetraacetic acid) (ATCC, PCS999003) containing 0.05% trypsin and 0.02% EDTA followed by Trypsin Neutralizing Solution (ATCC, PCS999004) according to the manufacturer’s recommendation. All reagents for cell cultures were purchased from Gibco (Life Technologies), unless otherwise stated.

For stimulations, monocytes were preincubated with 25 μM DMOG (Cayman, 71210) for 1 h. Recombinant HMGB1 (1 μg/mL) was added in X-Vivo 15 medium. HMGB1 was purified from *Escherichia coli* and extracted with Triton X-114 to remove any contaminating lipopolysaccharide (63). Endotoxin levels were monitored with the Limulus Amebocyte Lysate QCL-1000 kit (Lonza) and were undetectable (<0.1 EU/mL). Supernatant or cell lysates were harvested at the indicated times for further assays. Normoxic cells were maintained in a tissue culture incubator (21% O_2_ and 5% CO_2_) with an open water reservoir. Hypoxic cells were maintained in a hypoxia subchamber (2% O_2_ and 5% CO_2_). Cell viability was monitored by trypan blue exclusion (Lonza, 17-942E). All experiments were performed at least three times, in triplicate, unless otherwise stated in the figure legend.

### Plasmids and Transient Transfection.

Human monocytes (1 × 10^7^ cells) were transfected by Nucleofector kit (Lonza). HEK293T cells were grown overnight in 100-mm dishes to ∼70% confluency; cells were then transfected using Lipofectamine 2000 (Invitrogen) according to the manufacturer’s instructions. Cells were transfected with an HIF-1α–encoding plasmid (GeneCopoeia, EX-H2453-M13), the IRF5 promoter-driven Gaussia luciferase (Luc) reporter construct (GeneCopeia, HPRM33964-PG02), HIF-1α siRNA (Thermo Fisher, 4390826), or control siRNA (Thermo Fisher, 4390847). HIF-1α knockdown or HIF-1α overexpression efficiency was confirmed by qRT-PCR in each experiment (*SI Appendix*, Fig. S6). Three mutants of IRF5 promoter were made by GeneCopoeia through site-directed mutagenesis. SEAP encoding plasmid (GeneCopoeia, SEAP-PA01) was used for transfection efficiency. Dual-reporter promoter clone for IRF3 promoter was purchased from GeneCopoeia (HPRM38174-pEZX-PG04). After 24-h transfection, the supernatant was measured the luciferase and SEAP activities using a Secrete-Pair Dual Luminescence Assay Kit (GeneCopoeia, LF032).

Luciferase and alkaline phosphatase signals were measured on a Synergy Neo2 plate reader (BioTek).

### Lactate and IFNα Measurement.

Lactate levels in culture supernatant were measured by a Lactate Assay Kit (Biovision, K6071) and secreted IFNα was measured using a VeriKine Human IFNα ELISA kit (PBL assay science, 41100) according to the manufacturer’s instructions.

### qRT-PCR.

Total RNA was extracted from cells with a RNeasy kit (Qiagen, 74106), and complementary DNA (cDNA) was generated using an iScript cDNA synthesis kit (Bio-Rad, 1708891). Real-time PCR reactions were performed on a Light Cycler 480 II (Roche, 4729749001) using Light Cycler 480 probes Master (Roche, 04887301001). Primers for IRF5 (Hs00158114, Hs00973536), IRF3 (Hs1547282), TNFα (Hs00174128), IL-1β (Hs01555410), IFNα (Hs03044218), HIF-1α (Hs00153153), and HPRT1 (Hs99999909, Hs02800695) were purchased from Thermo Scientific. The genes of interest were normalized to the expression of housekeeping genes, and 2-^ΔCt^ calculated the relative expression.

### Western Blot.

Total cell lysates from monocytes (5 × 10^6^ cells) were prepared in 1x RIPA lysis buffer containing protease inhibitor and phosphatase inhibitor mixture (Invitrogen). Samples were boiled in Laemmli sodium dodecyl sulfate (SDS) buffer and subjected to immunoblot analysis in 4 to 12% gradient Bis-Tris SDS polyacrylamide gel electrophoresis (Invitrogen, NP0321) and subsequently transferred onto a polyvinylidene difluoride membrane (Applied Biosystems). The membrane was washed twice in phosphate-buffered saline (PBS) containing 0.05% Tween 20 (PBST), blocked by 5% skim milk in PBS for 1 h at room temperature, and then incubated overnight at 4 °C with rabbit anti-human IRF5 (1:1,000; Abcam, ab2932), anti–NF-κBp52 (1:250; Santa Cruz Biotechnology, sc-848), and mouse monoclonal anti–β-actin (1:2,000; Abcam, AC-15). After three washes in PBST, the membranes were incubated for 1 h in the respective secondary immunoglobulin G conjugated with infrared 680 or 800 (1:10,000; LICOR). After three washes in PBST, the membrane was scanned on a Sapphire Biomolecular Imager (Azure Biosystems). Densitometric analysis of band intensities was performed using NIH Image J software.

### Gene Expression Analysis by RNA-Seq.

Human monocytes (5 × 10^6^ cells, triplicates per donor) were stimulated with HMGB1 (1 μg/mL) for 4 h, washed, and subsequently harvested. For DMOG stimulation, cells (5 × 10^6^ cells, triplicates per donor) were preincubated with DMOG (200 nM) or dimethyl sulfoxide for 1 h prior to addition of HMGB1 (1 μg/mL). After 4 h, cells were harvested. Cell pellets were snap-frozen on dry ice and stored at −80 °C. RNA isolation, confirmation of RNA integrity, library preparation, and sequencing analysis were conducted at GENEWIZ, LLC. The quality of unstranded paired read files (FASTQ) were checked using FASTQC (v0.11.7, https://www.bioinformatics.babraham.ac.uk/projects/fastqc) then aligned to human GRCh38 genome using HISAT2 (v2.0.5). Aligned reads were sorted and indexed by SAMTOOLS (v1.3). Gene-level raw counts were calculated using featureCounts (v1.5.2) and normalized by the Trimmed means of M-values normalization method implemented in the edgeR package (64). The iGEAK was used to perform differential expression analyses after removing extremely low count genes (65). Differentially expressed genes between groups were identified by the cutoff criteria: |fold| > 2× and false discovery rate q < 0.05. DEG enriched pathways were detected using UP or DOWN (DN) genes based on adjusted *P* < 0.05. Raw and processed sequencing data files are available at Gene Express Ombibus (GEO accession number GSE162834).

### ChIP.

An Ab500 ChIP Kit (Abcam) was used according to the manufacturer’s instructions. Briefly, monocytes (5 × 10^7^ cells) or HEK293T cells (1 × 10^7^ cells) were centrifuged and fixed in 1.1% formaldehyde in PBS. Reactions were quenched with glycine and cells were washed in ice-cold PBS before lysis. Chromatin was sheared to ∼200- to 500-bp fragments using a sonicator at 4 °C. Chromatin was diluted and input chromatin was collected. Remaining chromatin was used for ChIP by adding 4 μg mouse anti–HIF-1α (Abcam; ab1) as the antibody of interest, 4 μg mouse anti-histone H3 (Abcam, ab1220) as a positive control, and mouse anti-GFP antibody (Abcam, ab1218) as a negative control. Antibodies were added for 12 h at 4 °C. Protein G Dynabeads (Thermo Fisher,10003) were used to precipitate protein/DNA complexes. Cross-linking was reversed by heating at 98 °C followed by Proteinase K addition and DNA purification. Samples were analyzed by PCR. The input DNA and immunoprecipitated DNA (20 ng) were amplified by PCR using primers encompassing the known HIF-1α binding sites on the IRF5 and IRF3 promoter regions. PCR products were detected on 2.0% agarose in 0.5× TBE buffer. The gel was prestained with SYBRsafe gel stain (Invitrogen, S33102) and the electrophoresis images were captured by Bio-Rad gel doc system (Bio-Rad). The following PCR primers were used for IRF5, forward: CTG​AGT​TGT​CCC​GCT​CTA​GC, reverse: CAC​CAA​CCC​AGG​AGA​GGT​AA and for IRF3, forward: GGC​CAC​TCC​TCT​TAC​CTA​GG, reverse: CAG​TCC​CTA​ACC​CTC​TCT​CTC.

### HMGB1 ELISA.

The quantification of human HMGB1 in the culture supernatant and cell lysates was determined using ELISA kits according to the manufacturer’s instruction (IBL International GmbH, ST51011). For measuring within the high-sensitivity range, 50 µL of diluents buffer was added to each microtiter plate followed by the addition of 50 µL of standard, positive control, and samples. The plate was covered with adhesive foil and incubated for 22 h at 37 °C. Each well was washed five times, and 100 µL of enzyme conjugate was added for another 2 h at room temperature. After washing five times, 100 µL of substrate solution was added then kept for 30 min at room temperature. The reaction was completed by adding 100 µL of stop solution, and the optical density was measured at 450 nm using a microplate reader (PerkinElmer, Victor3).

### Immunofluorescence Assay.

To determine the cellular localization of HMGB1, epithelial cells were cultured in LabTek II chambers (Nalgen) and fixed in 4% paraformaldehyde in PBS at room temperature for 30 min. The cells were then washed with PBS and incubated at 4 °C for 10 min with permeabilization buffer (PBS containing 0.1% Triton X-100). After blocking with 5% BSA in PBS for 1 h, cells were incubated with rabbit anti-HMGB1 antibody (Abcam, ab18256), followed by incubation for 1 h with Alexa Fluor 488–conjugated secondary antibody (Invitrogen). The cells were coverslip-mounted using a mounting medium containing DAPI (Invitrogen) and fluorescence was analyzed using a fluorescence microscope (Evos M7000). Images quantified using NIH Image J software.

### Statistics.

Statistical analysis and graphing were performed using GraphPad Prism 8. The variance of mean values between two groups was analyzed by the two-tailed *t* test for unpaired observations. Group differences were tested with one-way ANOVA followed by Tukey’s multiple comparisons test. *P* < 0.05 was considered significant.

## Supplementary Material

Supplementary File

## Data Availability

Sequencing data have been deposited in Gene Express Omnibus (accession no. GSE162834). All other study data are included in the article and/or *SI Appendix*.
