# Supplementary material for: HIF-1α is a negative regulator of interferon regulatory factors: Implications for interferon production by hypoxic monocytes
Source: Proc Natl Acad Sci U S A. 2021 Jun 9;118(26):e2106017118. doi: 10.1073/pnas.2106017118 (PMC8256008; doi:10.1073/pnas.2106017118)
Supplement: Supplementary File [file pnas.2106017118.sapp.pdf]

PNAS
www.pnas.org

Supplementary Information for

HIF-1α is a negative regulator of interferon regulatory factors: Implications for interferon production by hypoxic monocytes

Travis Peng[a], Shin-Yi Du[a], Myoungsun Son[a,b,1,2], and Betty Diamond[a,b,1]

[a]Center for Autoimmune Musculoskeletal and Hematopoietic Diseases, The Feinstein Institutes for Medical Research, Manhasset, NY, 11030; [b] Department of Molecular Medicine, Donald and Barbara Zucker School of Medicine at Hofstra/Northwell, Hempstead, NY,11549

[1]M.S. and B.D. contributed equally to this work.

[2]corresponding author: Myoungsun Son

E-mail: mson@northwell.edu

This PDF file includes:

Supplementary Methods

Figures S1 to S6

Supplementary Methods

Monocyte-derived macrophages culture

Peripheral blood mononuclear cells (PBMCs) were isolated from the blood of de-identified healthy donors (New York Blood Center). Monocytes were purified by a human monocyte enrichment kit (Stem Cell Technology, 19059). Monocytes ($1X10^5$ cells per well) were cultured in 24-well plates in X-Vivo 15 medium (Lonza, 04-418Q) with 50 ng/ml human recombinant M-CSF (Peprotech, 300-25). Fresh medium with M-CSF was added on day 3; cells were further cultured until day 6. Monocyte-derived macrophages (MDM) were stimulated with or without HMGB1 (1 μg/ml) for 4 hours. Hypoxic cells were maintained in a hypoxia subchamber (2% O2 and 5% CO_2). All experiments were performed with triplicate of each donor.

Human plasmacytoid dendritic cell (pDC) isolation

Human peripheral pDCs were purified with an EasySep kit using a negative selection to isolate pDCs (STEMCELL Technologies, 19062). The pDCs (3×10^4 cells per well) were placed in u-bottom 96-well plates and stimulated in X-Vivo 15 medium with or without HMGB1(1 μg/ml) for 24 hours in normoxic or hypoxic conditions.

IFNα measurement

Secreted IFNα was measured using a VeriKine Human IFNα ELISA kit (PBL assay science, 41100) according to the manufacturer's instructions.

Pyruvate and Succinate measurement

Succinate levels from cell lysates were measured by a colorimetric Succinate Assay kit (Abcam, ab204718). Pyruvate levels from cell supernatants were measured by a Pyruvate Assay Kit (Abcam, ab65342). Signals were measured on a Synergy Neo2 plate reader (BioTek).

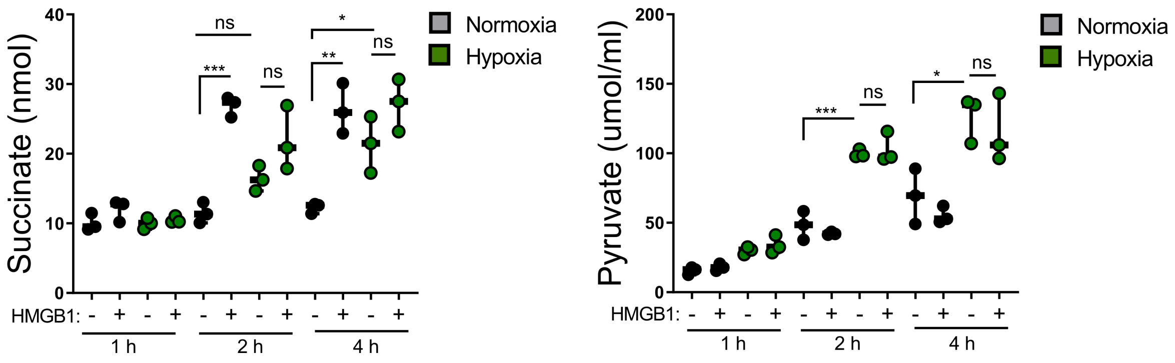

Supplementary Figure 1. Succinate and pyruvate are induced by hypoxia. Succinate is further increased by HMGB1. Human monocytes were exposed to 21% (normoxia) and 2% (hypoxia) oxygen with or without HMGB1 (1 μg/ml). Succinate measured from cell extracts at indicated time points. Pyruvate measured from cell supernatants. Floating bars (min to max); the line denotes median; each symbol represents an individual experiment performed in triplicate, *N*=3.

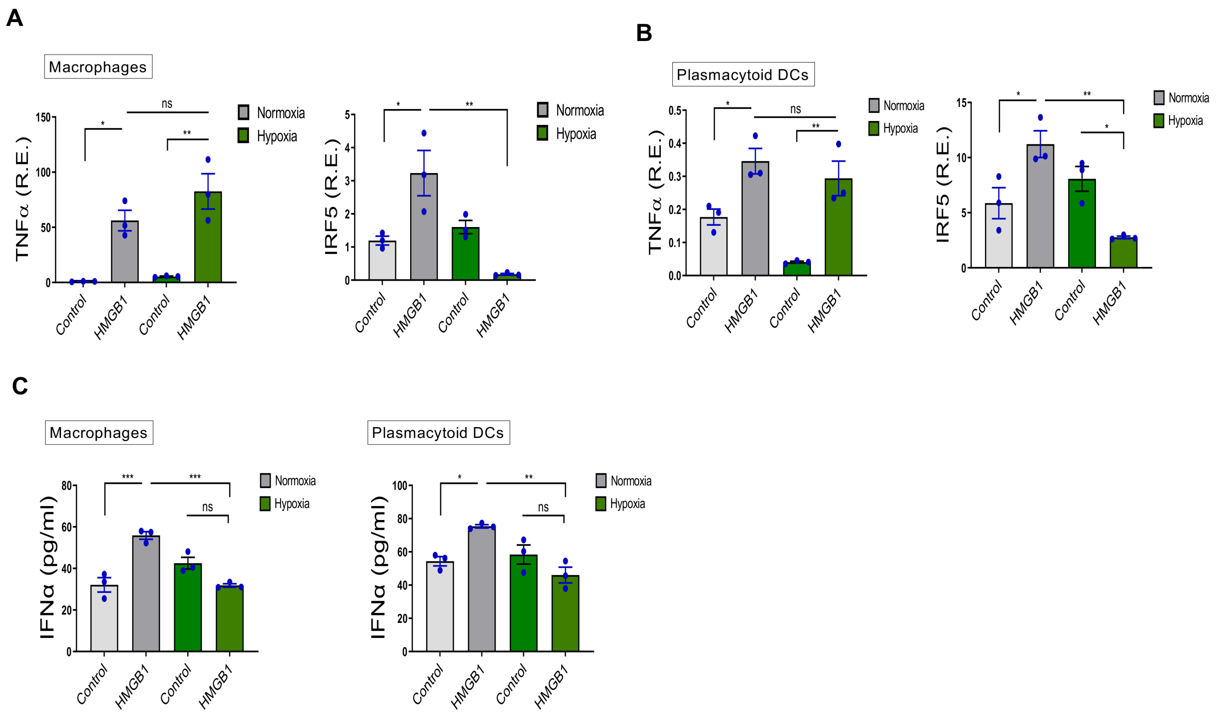

Supplementary Figure 2. HMGB1-induced IRF5 but not TNFα was reduced under hypoxia in human monocytes-derived macrophages (MDMs) and plasmacytoid DCs. (A) Human MDMs and **(B)** human plasmacytoid DCs were exposed to normoxia and hypoxia for 4 hrs with or without HMGB1. The expression of TNFα and IRF5 was analyzed by qRT-PCR. Each symbol represents an individual donor assayed in triplicate (mean ± SEM). **(C)** MDMs and plasmacytoid DCs were cultured in normoxic or hypoxic conditions for 24 hrs after which IFNα was measured in the supernatants by ELISA. Each symbol represents an individual donor assayed in triplicate (mean ± SEM). One-way ANOVA $^{*}P \leq 0.05$; $^{**}P \leq 0.01$; ns, $P > 0.05$.

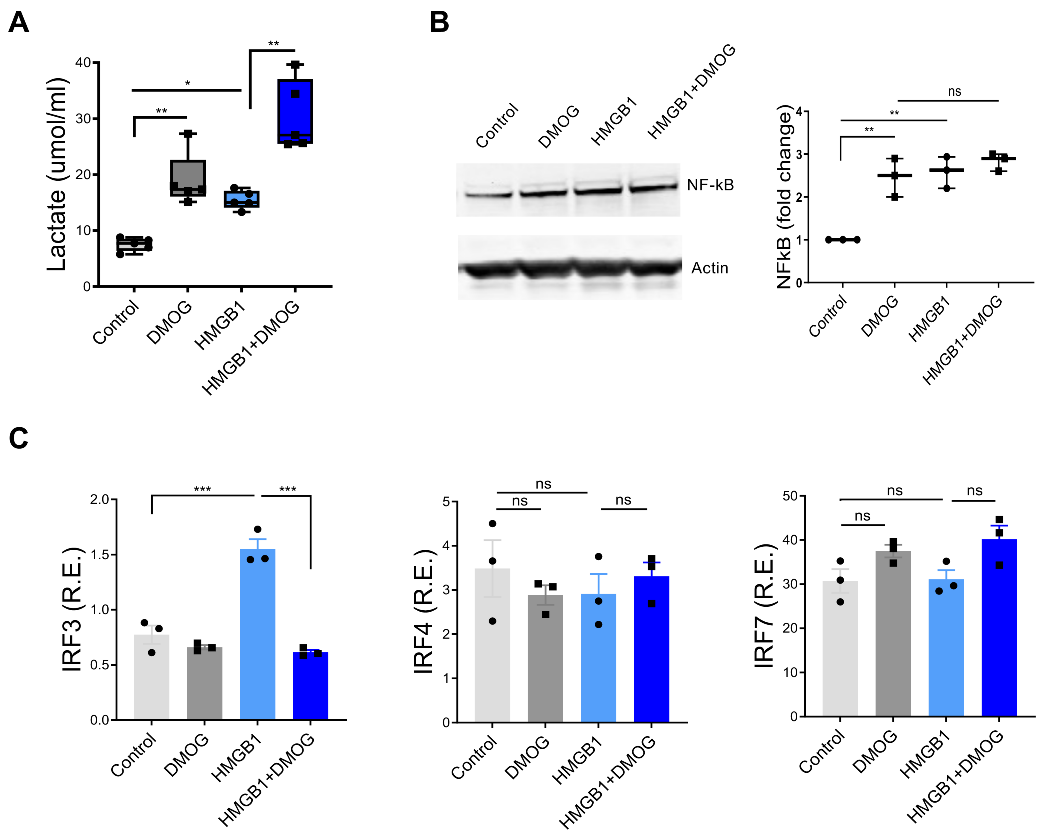

Supplementary Figure 3. DMOG mimics hypoxia. (**A**) Human monocytes were pre-incubated with DMOG (25 μM) for 1 hr, then stimulated with HMGB1 (1 μg/ml) for 4 hrs. Lactate, measured in the cell supernatant, was significantly increased by DMOG and HMGB1 exposure. Floating bars (min to max); the line denotes median; each symbol represents an individual experiment in triplicate, *N*=5. (**B**) Western blots analyzed NF-κB in cells under resting and HMGB1 stimulated conditions, with or without DMOG. One representative experiment of 3 independent experiments is shown (left). Fold changes compared to control were calculated by densitometry (right). Each symbol represents an individual experiment, *N*=3. (**C**) IRF3, IRF4 and IRF7 mRNA. Mean ±SEM, *N*=4. One-way ANOVA *$P \leq 0.05$; **$P \leq 0.01$; ***$P \leq 0.001$; ns, $P > 0.05$.

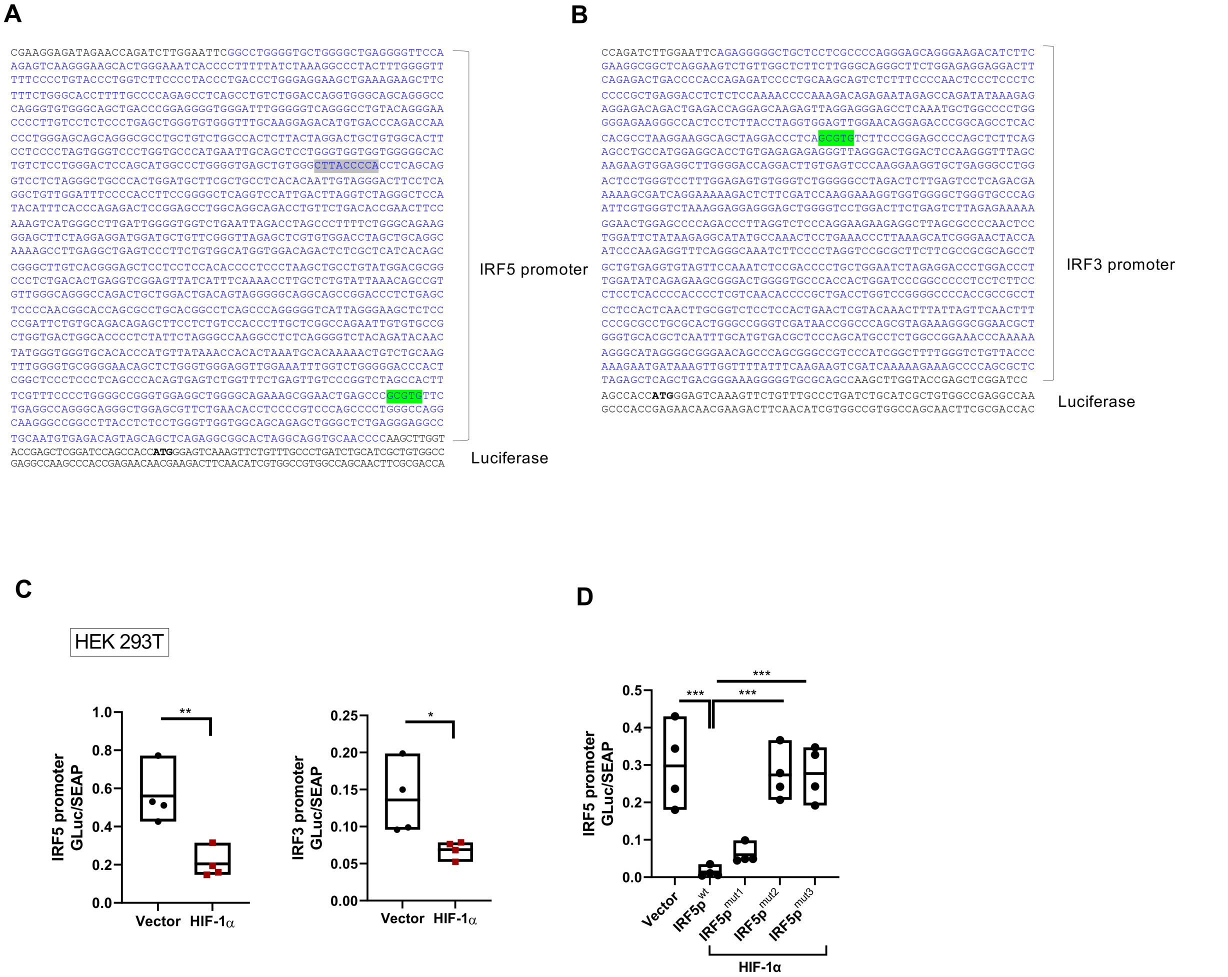

Supplementary Figure 4. HIF-1α requires the hypoxia-response element (HRE) sequence to bind the IRF5 and IRF3 promoters. (A) Sequence of the IRF5 promoter (GeneCopeia, HPRM33964-PG02) (**B**) Sequence of the IRF3 promoter (GeneCopoeia, HPRM38174-pEZX-PG04). (**C**) IRF5 or IRF3-promoter Guassiluciferase (Gluc) constructs and a HIF-1α encoding plasmid were transfected into HEK293T cells. Gluc and secreted alkaline phosphatase (SEAP) activity was measured. The ratio of Gluc to SEAP was calculated. Floating bars (min to max); the line denotes mean; each symbol represents an individual experiment. Unpaired *t*-test. *$P \leq 0.05$; **$P \leq 0.01$. mean ±SEM. *N*=4. (**D**) HIF-1α binds the HRE site in the IRF5 promoter. The assay was performed in HIF-1α overexpressing HEK293T cells. Wild type IRF5 promoter ($IRF5p^{wt}$) and IRF5 promoter mutant 1 ($IRF5p^{mut1}$) were suppressed by HIF-1α but not HRE mutants of the IRF5 promoter ($IRF5p^{mut2}$ and $IRF5p^{mut3}$). Floating bars (min to max); the line denotes mean. *N*=4. One-way ANOVA. *$P \leq 0.05$; **$P \leq 0.01$; ***$P \leq 0.001$; ns, $P > 0.05$.

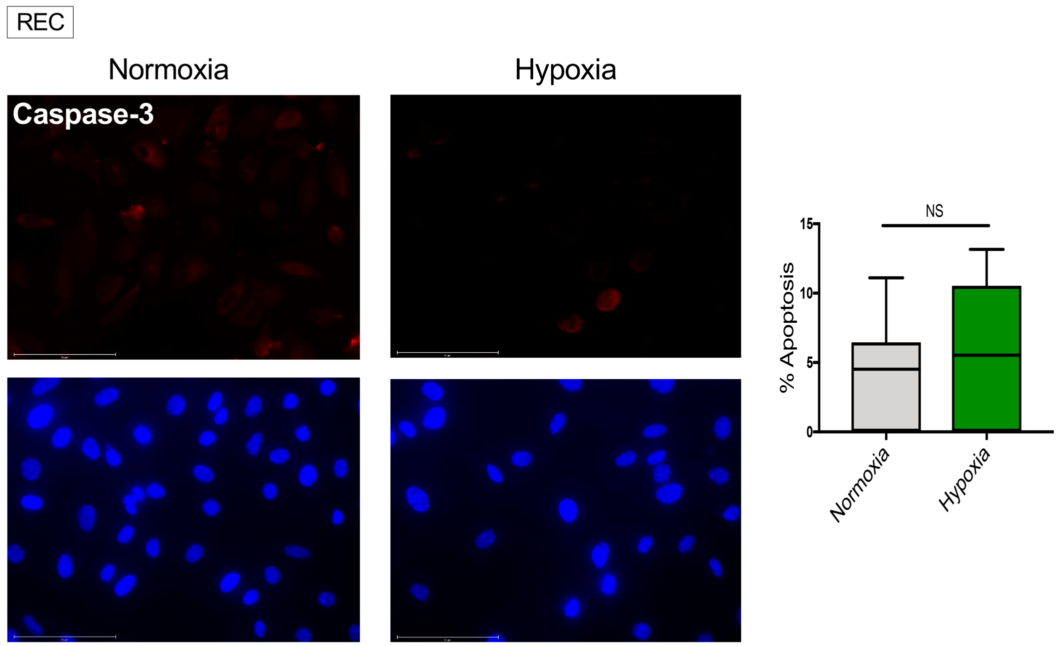

Supplementary Figure 5. Viability of renal tubular epithelial cells (REC) is similar in normoxic and hypoxic conditions (48 hrs). Apoptotic cells were detected by PE Active Caspase-3 Apoptosis Kit (red, BD Pharmingen, 550914). Nuclei were labeled with DAPI (blue). The percentage of apoptotic cells was counted in three experiments. Mean ±SEM. Unpaired *t*-test. ns, $P > 0.05$.

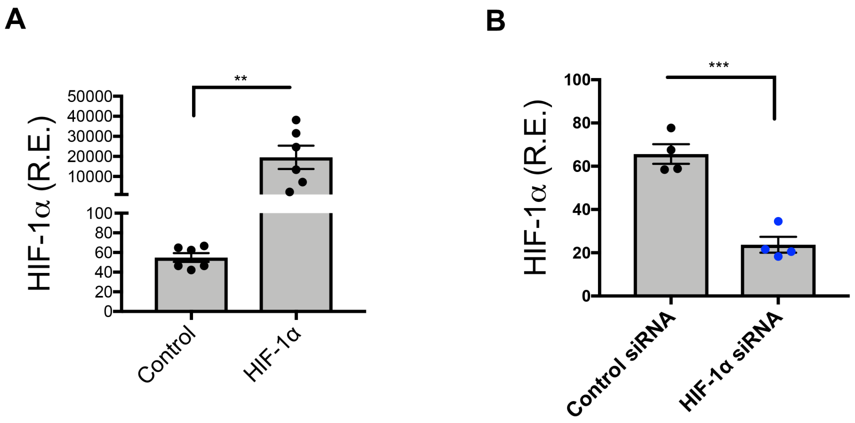

Supplementary Figure 6. HIF-1α overexpression and knockdown efficiency was determined on day 1 after transfection. HIF-1α levels were determined by qRT-PCR. Each symbol represents an individual experiment. Mean ±SEM. Unpaired *t*-test. *$P \leq 0.05$; **$P \leq 0.01$.
